# Using biomarkers to allocate patients in a response-adaptive clinical trial

**DOI:** 10.1080/03610918.2021.2004420

**Published:** 2021-11-25

**Authors:** H. Jackson, S. Bowen, T. Jaki

**Affiliations:** a Lancaster University, Lancaster, UK; b Quanticate, Wilmslow, UK; c University of Cambridge, Cambridge, UK

**Keywords:** Response adaptive clinical trial, Biomarker, Patient benefit, Personalized medicine

## Abstract

In this paper, we discuss a response adaptive randomization method, and why it should be used in clinical trials for rare diseases compared to a randomized controlled trial with equal fixed randomization. The developed method uses a patient’s biomarkers to alter the allocation probability to each treatment, in order to emphasize the benefit to the trial population. The method starts with an initial burn-in period of a small number of patients, who with equal probability, are allocated to each treatment. We then use a regression method to predict the best outcome of the next patient, using their biomarkers and the information from the previous patients. This estimated best treatment is assigned to the next patient with high probability. A completed clinical trial for the effect of catumaxomab on the survival of cancer patients is used as an example to demonstrate the use of the method and the differences to a controlled trial with equal allocation. Different regression procedures are investigated and compared to a randomized controlled trial, using efficacy and ethical measures.

## 1 Introduction

Randomized controlled trials (RCTs) are the approach most often used in Phase II-III clinical trials. In RCTs the probability of being assigned the experimental treatment and the control (placebo or standard of care) is typically fixed throughout the trial and often equal between each treatment arm. Hence, each intervention is given to a similar number of patients (Villar, Bowden, et al. 2015). This leads to the trial having large power. However, if it emerges before the end of the trial that one treatment is more effective, it would be sensible, from a patient’s perspective, to allocate the remaining patients to the estimated best treatment, a feature not included in traditional RCTs.

Using equal allocation makes sense in situations, where only a small proportion of the patient population will enter the clinical trial as many patients outside the trial will benefit from its results and the high power ensures this happens quickly. However, performing an RCT in a rare disease trial could mean that a large proportion of the general patient population is entered into the clinical trial, stated by Williamson et al. (2017). Under these circumstances, there should be a larger emphasis on the benefit to the trial population than on the general population. For this reason, response-adaptive randomization (RAR) is a design that is particularly suitable for clinical trials in rare diseases.

RAR trials use information from previous patients within the study, to decide which treatment is allocated to the next patient or next group of patients. The treatment allocation probability is varied to favor the estimated best treatment. This increases the number of successful outcomes in patients, as explained by Cheung et al. (2006). RAR designs intend to balance learning (identifying the best treatment, if there is one) and earning (treating as many patients as effectively as possible). They often include an initial ‘burn-in’ period where a small number of patients are allocated to each treatment with a fixed ratio (normally 1:1) (Thorlund et al. 2018). This ensures enough data is initially accumulated to allow an accurate initial estimation of which treatment is best.

RAR designs have been used in multiple clinical trials including a phase II trial comparing Z-102 with placebo in patients with rheumatoid arthritis (NCT01369745). There are, however, a few reasons why some medical professionals do not wish to use a RAR design.

Common draw backs of using RAR designs include suffering from low power and not handling time trends well (Proschan and Evans 2020). However, it is important to remember the large variety of RAR designs and their vast subclasses. It is very hard to generalize these issues for all RAR designs. In certain cases, Robertson et al. (2020) show, that some RAR designs have higher power than fixed randomization. In addition, it has been noted that certain RAR designs, particularly those which protect the allocation of patients to the control treatment, are not largely affected by time trends (Robertson et al. 2020). Another assumption of many RAR designs, including the Gittins index (Chakravorty and Mahajan 2014), forward-looking Gittins index (Villar, Wason, et al. 2015), and randomized play-the-winner (Rosenberger 1999), is they assume each patient in the trial will react in the same way if given the same treatment. In an era of personalized medicine, we know that this is not always the case. Some people have certain characteristics which can cause them to react differently to the same treatment.

We can use these patient characteristics (also known as covariates or biomarkers) in order to allocate patients to a certain treatment in a RAR clinical trial (Villar and Rosenberger 2018). If one treatment is identified to work better on a patient with certain biomarkers, then the probability of allocating that treatment to the next patient can be adjusted depending on their biomarkers. This leads to improved outcomes for patients within the trial. A couple of different such covariate adjusted response adaptive (CARA) designs have been proposed, see Villar and Rosenberger (2018) and Rosenberger, Vidyashankar, and Agarwal (2001), who focus on methods for binary patient outcomes. In particular, Thall and Wathen (2005) describe a multi-stage adaptive design, which uses a Bayesian framework to adaptively randomize patients to two treatments using their covariate values, and includes rules for stopping the trial early at an interim analysis. They use a probability model which accounts for the multi-stage treatment and baseline covariates. This method focuses on binary covariates and categorical patient responses only. Qiao, Ning, and Huang (2019) use a Bayesian logistic regression model to assess the association between the patients’ outcome and their covariates. They then calculate the assignment probabilities, using the predicted probability of each treatment producing a success in the next patient. This design is only applicable when the patient’s outcome is binary. Sverdlov, Rosenberger, and Ryeznik (2013) investigate two CARA designs. One method focuses on obtaining the optimal allocation to minimize the total expected hazard in a trial and the other approach looks at a design to optimize a utility function which combines inferential and ethical criteria in a weighted fashion. These designs are used in the survival setting only. A CARA design was used in the phase II, multicenter trial, I-SPY 2, to screen experimental designs for breast cancer (Rugo et al. 2016). This trial, among others, has shown RAR trials should not be banished to theory and are feasible in application.

The rest of the article is organized as follows. We describe a clinical trial, where the RAR method could be used in Sec. 2. In Sec. 3, we explain our method. The main contribution of this article is in Sec. 4 and 5, where we evaluate the method in two simulation studies. The simulation study in Sec. 4 includes a single, continuous biomarker and in Sec. 5, the simulation study is evaluated for the case study described in Sec. 2. Finally, we note our conclusions and explore further work in Sec. 6.

## 2 Case study

The effect of catumaxomab in the treatment of malignant ascites was investigated by Heiss et al. (2010), in a phase II/III study (NCT00836654). This study comprised a population of 245 patients, but screening data was only available for 233 patients. *N*_0_ = 83 patients were given the control treatment and *N*_1_ = 150 patients were given catumaxomab. It showed the treatment of malignant ascites due to different epithelial cancers was improved by the use of catumaxomab plus paracentesis. This treatment prolonged puncture-free survival (PuFS) when compared with paracentesis alone (median, 46 vs 11 days, *P* < 0.0001). In the original study PuFS was the primary endpoint and overall survival (OS) was a secondary endpoint. The treatment catumaxomab versus paracentesis alone also showed an improvement in OS (median, 72 vs 68 days, *P* = 0.0846), although this was not found to be statistically significant.

Catumaxomab was further investigated by Heiss et al. (2014) in regards to the effect of biomarkers on the patient’s outcomes. An exploratory post hoc analysis was performed on the impact of several biomarkers. The two biomarkers: relative lymphocyte count (*RLC*) and Karnofsky Index (*KI*) were shown to have a significant impact on the OS of the patients given catumaxomab.

In a subgroup analysis the trial population was split into two subgroups depending on their *RLC* value. In the subgroup of patients with an *RLC* > 13%, 59 were given the control and 100 were given catumaxomab. In this subgroup, catumaxomab was associated with a longer OS when compared with the control, 109 days compared with 68 days, respectively (*P* = 0.0072). In patients with an *RLC* ≤ 13%, 24 patients were given the control and 50 patients were given catumaxomab. In this subgroup, there was no significant difference in the median OS between the two treatment groups, (*P* = 0.2561) (Heiss et al. 2014).

In another subgroup analysis the trial population was also split into two subgroups depending on their *KI* value. In the subgroup of patients with a *KI* ≥ 70%, 71 were given the control and 129 were given catumaxomab. In this subgroup, catumaxomab was associated with a longer OS when compared with the control, 84 days compared with 62 days, respectively (*P* = 0.0053). In patients with a *KI* < 70%, 12 patients were given the control and 21 patients were given catumaxomab. There was no significant difference in median OS between the two treatments in this subgroup. The effects of the biomarkers on the treatment were calculated using Cox proportional hazards models (Heiss et al. 2014).

The ability to predict the response to cancer therapy is an important area of clinical research, and there have been many attempts to identify biomarkers which correlate with a positive outcome in a patient (Heiss et al. 2014). Therefore, these biomarkers could be used to choose the patients who will benefit most from the treatment and hence, can guide treatment decision making for personalized medicine.

## 3 A response adaptive design with biomarkers

In a clinical trial, assume we have *K* ≥ 2 treatments and a total of N patients. Patients arrive into the trial sequentially, such that the outcome of patient *n* is known before patient *n* + 1 enters the trial. For each patient *n* ∈ {1, *N*} assume further that a biomarker value *x_n_* is observed at baseline. In general, any covariate could be used, but in our application we will use the terms bio-marker and covariate interchangeably. In the design, this biomarker, *x_n_* along with information from previous patients, is used to determine which treatment that patient should be allocated to.

For each treatment *k* ∈ {1, *K*} we model the random outcome *Y_n, k_* of patient *n* as a function of each patient’s biomarker value, thus, *Y_n, k_* = *f_k_*(*x_n_*). No assumption on the form of the function is made. This outcome could be binary, such as the treatment curing the patient or not, integer valued, such as the number of epileptic fits in six months, continuous, such as the percent change in bone mineral density at the Lumbar Spine of a patient, or it could be the survival time of a patient (Yang and Zhu 2002). We assume only one treatment is given to each patient and the observed outcome denoted by y_n_, _k_ for patient n who is given treatment *k*, is known immediately.

An allocation rule, *A*, must be found such that *I*_1_, *I*_2_,…,*I_N_* represents the treatments allocated to patients 1, 2,…,*N*, in order to maximize the number of patients producing a successful outcome. The mean outcome in patient *n*, with given biomarker *x_n_* is *f_I_n__* (*x_n_*) for *n* ≥ 1 (Yang and Zhu 2002).

The most favorable allocation policy A* is when the treatments that are chosen match the optimal choice of treatment k*(*x*_1_),…, k*(*x_N_*). Here, k*(*x_n_*) is the treatment which produces the best outcome for patient *n*. This policy A* yields the optimal total outcome $\Sigma_{j=1}^{N}f_{k*\left(x_{j}\right)}\;\left(x_{j}\right)$ (Yang and Zhu 2002). Thus, the random variable R measures the performance of the allocation rule *A* relative to the ideal allocation rule A*,

$$R\left(A\right)=\frac{\Sigma_{j=1}^{N}f_{I_{j}}\left(x_{j}\right)}{\Sigma_{j=1}^{N}f_{k*\left(x_{j}\right)}\left(x_{j}\right)}.$$

If we knew these functions *f_k_*(*x*), when a new patient *n* arrives into the trial we could find the assumed outcome of that patient for each treatment (given their biomarker value), *Y_n, k_* = *f_k_*(*x_n_*) and assign patient *n* to the treatment with the best assumed outcome, max_1≤*k*≤*K*_(*Y_n,k_*).

In practice, we do not know *f_k_*(*x_n_*) nor do we know it’s functional form. Consequently, we will use a flexible regression procedure and the outcomes of all previous patients who were given treatment *k*, ***y_n−1,k_***, to estimate it with, ${\hat{f}}_{k,y_{n-1,k}}\left(x_{n}\right)$. We use the same regression method to estimate each function, ${\hat{f}}_{k,y_{n-1,k}}\left(x_{n}\right)$, for each treatment *k* ∈ {1, *K*}.

Putting this together, there are two main parts of our method:

- Non-parametric estimation of each function *f_k_*.
- Allocation rule to balance learning which treatment is best and choosing the estimated best treatment.

The full algorithm (altered from what was proposed by Yang and Zhu (2002)) for the biomarker adjusted RAR procedure is stated below.

**Algorithm 1: RAR Algorithm**

1. Allocate each of the first *L* × *K* patients who enter the trial to the K treatments with equal probability.
2. Given we know the biomarker *x_n_* of the next patient *n*, use the regression procedure and information from previous patients to find the treatment with the best estimated outcome $\left(\max_{1\leq k\leq K}\left\{{\hat{f}}_{k,y_{n-1,k}}\;\left(x_{n}\right)\right\}\right)$.
3. Select the best estimated treatment with probability 1 – (*K* – 1)*π_n_* and select the other treatments) with probability *π_n_*.
4. Use the observed outcome of patient *n, y_n, k_* and biomarker *x_n_* to update the estimate ${\hat{f}}_{I_{n}}\left(x\right)$.
5. Repeat steps 2-4 for the next patients *n* + 1, *n* + 2,…, *N*.

The first step is the ‘burn-in’ period, where *L* patients are assigned to each of the *K* treatments (Lewis et al. 2013). After the burn-in period, the regression method is used to estimate the best treatment for the next patient. They initially have *L* points to use to first estimate each treatment’s outcome in patient (*L* × *K*) + 1, *Y*_*k*,(*L*×*K*)+1_∀*k* ∈ {1, *K*}. As more patients enter the trial, the regression method has more information and their estimate of the best treatment should become more accurate. The sequence *π_n_* allows us to control the probability of each patient receiving their estimated best treatment. A full description of the regression procedures investigated in this work are detailed in Appendix A.

## 4 Simulation

We compare the proposed approach, using different regression methods (described in Appendix A) with equal fixed randomization (FR), in a number of two treatment trial scenarios via simulations. The detailed implementation, such as tuning parameters, for each regression method are described in Appendix C. A uniformly distributed biomarker *x_n_* ∈ [–100,100], e.g., weight change measured in pounds and a continuous outcome, e.g., percent change in bone mineral density, are used.

In the following scenarios we model each patient’s outcome as a function of the patient’s bio-marker value plus a random error term, *Y_n, k_* = *f_k_*(*x_n_*) + *ϵ_n_*. The function used changes for each of the treatments, in each of the scenarios we investigate. In addition, the random error term *ϵ_n_* is dependent on the outcome, where a larger outcome, gives an increase in variability. We investigated a constant random error term as well, however, it produced qualitatively similar results and thus, we omit it from our comparisons.

Here, we are using simulations with only one biomarker. All the regression methods investigated can be adapted, to use multiple biomarkers to predict which treatment will be best for the patients who enter the trial. Random forests in particular can handle more complex cases. However, as all methods should work in this simple one biomarker simulation we use it as a starting point.

We use a simulation of size 10 000 for all regression methods except random forest, where 1 000 simulations are used due to computational constraints. We use trial sizes of *N* = 40, *N* = 80 and *N* = 120 to reflect that we are considering the context of rare disease trials.

The trial begins with the burn-in period, where the first 10 patients are randomized to the two treatments in a 1:1 ratio. From the 11th patient onwards, each patient n is assigned to their estimated best treatment with probability 1 – *π_n_*. We define *π_n_* as a linearly decreasing sequence from *π*_10_ = 0.5 to *π_N_* = 0.1. In this way, as we learn more about the treatments and get more confident in our estimation of which treatment is ‘best’, we are more likely to choose the next patient’s ‘best’ treatment. However, there will always be at least a 0.1 probability of allocating a ‘lesser’ treatment to the next patient.

### 4.1 Scenarios

**Figure 1:**
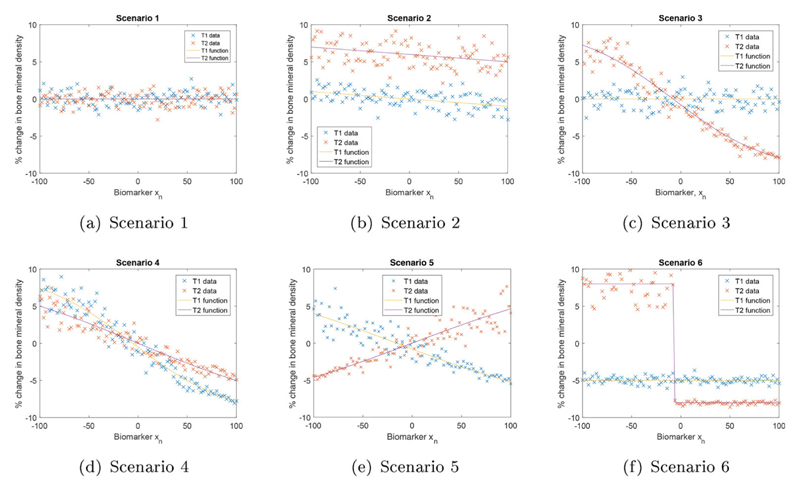
Simulation scenarios.

The performance of the proposed approach has been investigated under a range of different scenarios. Figure 1 displays the relationships between the patient’s biomarker and their outcome for both treatments and the underlying functions are given in Table B1 in Appendix B.

In addition, Figure 1 shows the random error term *ϵ_n_* is dependent on the mean outcome of patient *n, f_k_*(*x_n_*). If the outcome changes due to the patient’s biomarker, then when the outcome is small, the random error term is small and when the outcome is large, the random error term is also large. If the outcome of one treatment is independent of the biomarker value, the error term for this treatment is equal to the error size of the other treatment where the two treatment outcomes cross.

In scenario one, both treatments produce the same percent change for every patient, regardless of their biomarker value. Scenario two investigates the presence of a prognostic marker. A prognostic marker is a clinical or biological characteristic that gives information on a patient outcome irrespective of which treatment they are given, explained by Sechidis et al. (2018). If a biomarker is prognostic then, the outcome of both treatments increases (by a similar amount) as a patient’s biomarker changes.

Scenario three investigates the presence of a predictive marker. A predictive marker is defined by Sechidis et al. (2018) as a clinical or biological characteristic that suggests the benefit to the patient from the treatment, in comparison to their state at baseline. If a biomarker is predictive then, the outcome from a treatment is better if a patient has a certain biomarker value. In scenario three, which treatment is best changes at a biomarker value of *X* = – 8.

Scenario four investigates the presence of a marker that is predictive and prognostic. If a biomarker is predictive and prognostic then, the outcome of both treatments will increase as the patient’s biomarker value changes, however, the rate at which the outcome changes will differ for different treatments. In scenario four, the two treatments cross at *X* = – 11.

The last two scenarios also investigate the presence of a predictive marker. The two treatments cross at a biomarker value of *X* = – 8 in both scenario five and six. In scenario six, treatment one is not affected by the biomarker value of the patient and treatment two is a step function. There is no gradual decrease in the outcome as the patient’s biomarker value increases.

### 4.2 Performance measures

In order to compare the different regression methods we use the ethical performance of each design, as well as their type I error and power.

- **Proportion of patients who are allocated to the best treatment**, (here ‘best’ is interpreted as the treatment with the highest outcome in each individual patient). This is an ethical measure which we want to maximize.
  In FR we know this value will be roughly 0.5. In our RAR method this measure should always be above 0.5, as long as the estimation of which treatment is best is accurate.
- **Type I error**
  - Overall one-sided type I error, α_1_, is the probability you incorrectly identify the experimental treatment produces a larger outcome than the control treatment over the whole bio-marker range, when it does not. Here, we include all patients in the trial, in this calculation. We choose α_1_ = 0.025.
  - Overall two-sided type I error, α_2_, is the probability you incorrectly identify a difference between the two treatments over the whole biomarker range, when a difference does not exist. Here, we include all patients in the trial, in this calculation. We choose α_2_ = 0.05.
  - Due to the biomarker value affecting which treatment is best for a patient, we will also investigate both the one-sided and two-sided type I error for specific biomaker subsets of the data. We will investigate type I error for patients with biomarkers *x_n_* ≥ 0, thus, we only include the patients in the trial who have biomarker values *x_n_* ≥ 0, in this calculation. Additionally, we investigate type I error for patients with biomarkers *x_n_* < 0, hence, we only include the patients in the trial who have biomarker values *x_n_* < 0, in this calculation. This reflects the situation where we have prior knowledge suggesting that at biomarker value *x_n_* = 0, the better treatment changes. We also investigate the type I error for patients with biomarkers *x_n_* ≥ *X* and *x_n_* < *X*, where, *X* is the actual biomarker value where the best treatment changes, to provide a bench mark for the performance. We do not investigate the type I error, with an estimated crossing point, $\hat{X}$, as it would not give a good approximation of the type I error, due to selection bias, (Bauer et al. 2010). The one-sided type I error in these subgroups is chosen to be 0.0125 and the two-sided type I error in these subgroups is 0.025.
- Power
  - Overall one-sided power, (1 – β_1_), is the probability you correctly identify the experimental treatment produces a larger outcome than the control treatment over the whole bio-marker range, when it does. Here, we include all patients in the trial, in this calculation.
  - Overall two-sided power, (1 – β_2_), is the probability you correctly identify a difference between the two treatments over the whole biomarker range, when a difference does exist. Here, we include all patients in the trial, in this calculation.
  - Due to the biomarker value affecting which treatment is best for a patient, we will also investigate both the one-sided and two-sided power for specific biomaker subsets of the data. We will explore power for patients with biomarkers *x_n_* ≥ 0, *x_n_* < 0, *x_n_* ≥ *X* and *x_n_* < *X*. For each of these calculations we only include patients in the trial who have biomaker values, *x_n_* ≥ 0, *x_n_* < 0, *x_n_* ≥ *X* and *x_n_* < *X*. Here, *X* is the actual biomarker value where the best treatment changes.

### 4.3 Results

#### 4.3.1 Ethical measure

**Figure 2:**
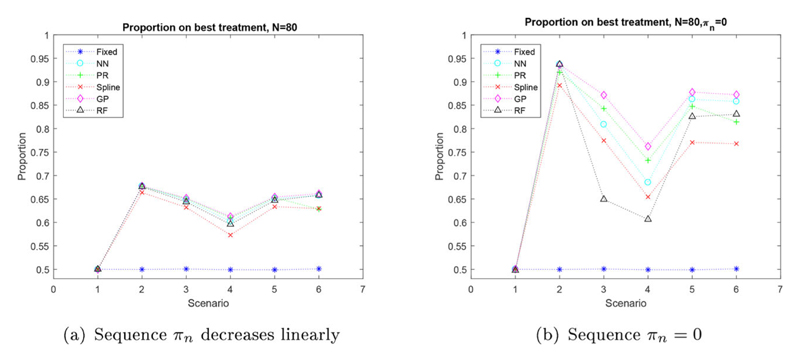
Simulated proportion of patients on their best treatment, when the sequence, *π_n_*, is a linear decrease (a) and when the sequence, *π_n_*, is equal to zero (b) for six scenarios with a sample size of *N* = 80.

The proportion of patients who receive the best treatment for them as an individual, for each scenario with a sample size of *N* = 80 is shown in Figure 2. The left plot shows the proportion of patients who are given their best treatment, when the sequence, *π_n_*, is used. The right plot gives the results for proportion of patients assigned to their best treatment when the sequence is equal to zero, *π_n_* = 0∀*n* ∈ {11, *N*}, and the estimated best treatment is allocated to the next patient with a probability of 1, after the burn-in period. It is apparent that all regression methods assign a higher proportion of patients to their best treatment, than the 50% we see using equal fixed randomization. The plot in Figure 2 also highlights that both the scenario and the regression method used, affects the proportion of patients who are allocated their best treatment. The regression methods are best at detecting which treatment is best in scenario two, due to the best treatment not being affected by the patient’s biomarkers. Other than scenario one, scenario four has the smallest proportion of patients given their best treatment. This is due to the small difference between the treatment outcomes for all patient biomarker values. For the majority of scenarios three regression methods perform best: Gaussian processes, polynomial regression and the nearest neighbor method. These methods assign a maximum proportion of 0.6774 patients to their best treatment, in scenario two. Splines tend to produce the smallest proportion of patients assigned to their best treatment.

As sample size increases, we see in the supplementary materials that the proportion of patients assigned to their best treatment also increases. When there are more patients in the trial there is more information and hence, the regression methods should be better at detecting which treatment is best, supported by Jenkins and Quintana-Ascencio (2020).

The proportion of patients who receive the best treatment for each scenario with a sample size of *N* = 80, where the sequence is equal to zero, *π_n_* = 0∀*n* ∈ {11, *N*}, is shown in Figure 2b. By keeping the sequence equal to zero, *π_n_* = 0, there is no randomization after the burn-in period and the patients always receive the treatment which is estimated to be best for them. In this method we can actually assign many more patients to their best treatment. We see in scenario two an increase of roughly 0.2 in proportion of patients who are assigned their best treatment, between Figure 2a, b. The other scenarios do not see such an extreme increase, but all scenarios and all regression methods (with the exception of the random forest in scenarios three and four) do produce an increase in proportion of patients allocated to their best treatment, when the sequence is equal to zero, *π_n_* = 0∀*n* ∈ {11, *N*}.

#### 4.3.2 One-sided type I error and power

**Figure 3:**
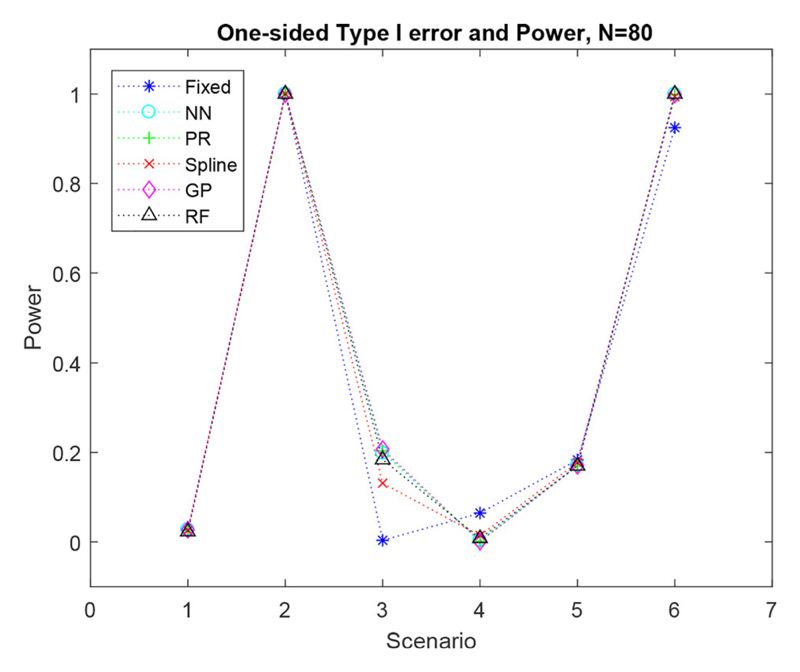
Simulated one-sided type I error and overall power for six scenarios with a sample size of *N* = 80.

The FR method, with equal allocation, has always been thought to give large power, as it assigns equal numbers of patients to each treatment. However, this is not always the case. Figure 3 represents the overall type I error for scenario one and overall one-sided power for scenarios two-six with a trial size of *N* = 80 patients.

For scenario two, treatment two (the experimental treatment) is best for all biomarker values, hence, the power for all methods is high. For scenario three, due to the crossing point being at *X* = – 8, treatment one (the control treatment) is best for the majority of biomarker values. The response adaptive methods adjusts for this and still allocates most patients to their best treatment, leading to a better ability to detect a difference between the arms. For scenarios four and five, due to the crossing point being negative, for a small majority (55%) of biomarker values treatment two is best. In scenario four the difference is very small, hence, the power of all methods is low. The FR has power of roughly 0.2 for scenario five, as the difference between the two treatments is slightly larger. All the regression methods produce a very similar power to the FR method for scenario five. In scenario six all the regression methods produce slightly higher power than the FR.

As expected with increasing sample size the power for all methods increase, although the order of performance remains the same (see the supplementary materials).

In scenario three and scenario six the regression methods produce a higher power than FR in Figure 3. This is due to the majority of patients who are assigned to treatment two in the regression methods, being biomarker negative and thus, increasing the overall average outcome. Whereas, the patients assigned to treatment two by the FR design are a mixture of patients with high and low biomarker values. Therefore, the mean outcome of patients on treatment two will be closer to the mean outcome on treatment one for FR, when compared with the regression methods. This is why the one-sided power of the adaptive designs is higher than the one-sided power of the FR design for scenario three. A similar outcome is observed for scenario six.

However, we cannot only look at the overall power, as many of the scenarios have a crossing point, *X*, where the best treatment changes. In the scenarios investigated, splitting the data at *x_n_* = 0 produced a similar power to that produced when the data was split at the actual crossing point. Thus, estimating the crossing point at *x_n_* = 0 is a good approximation to the actual crossing point. However, if the actual crossing point is further away from zero at for example, *X* = – 55, then splitting the data at zero will not give a good estimate of the power produced when the data is split at the actual crossing point.

**Figure 4:**
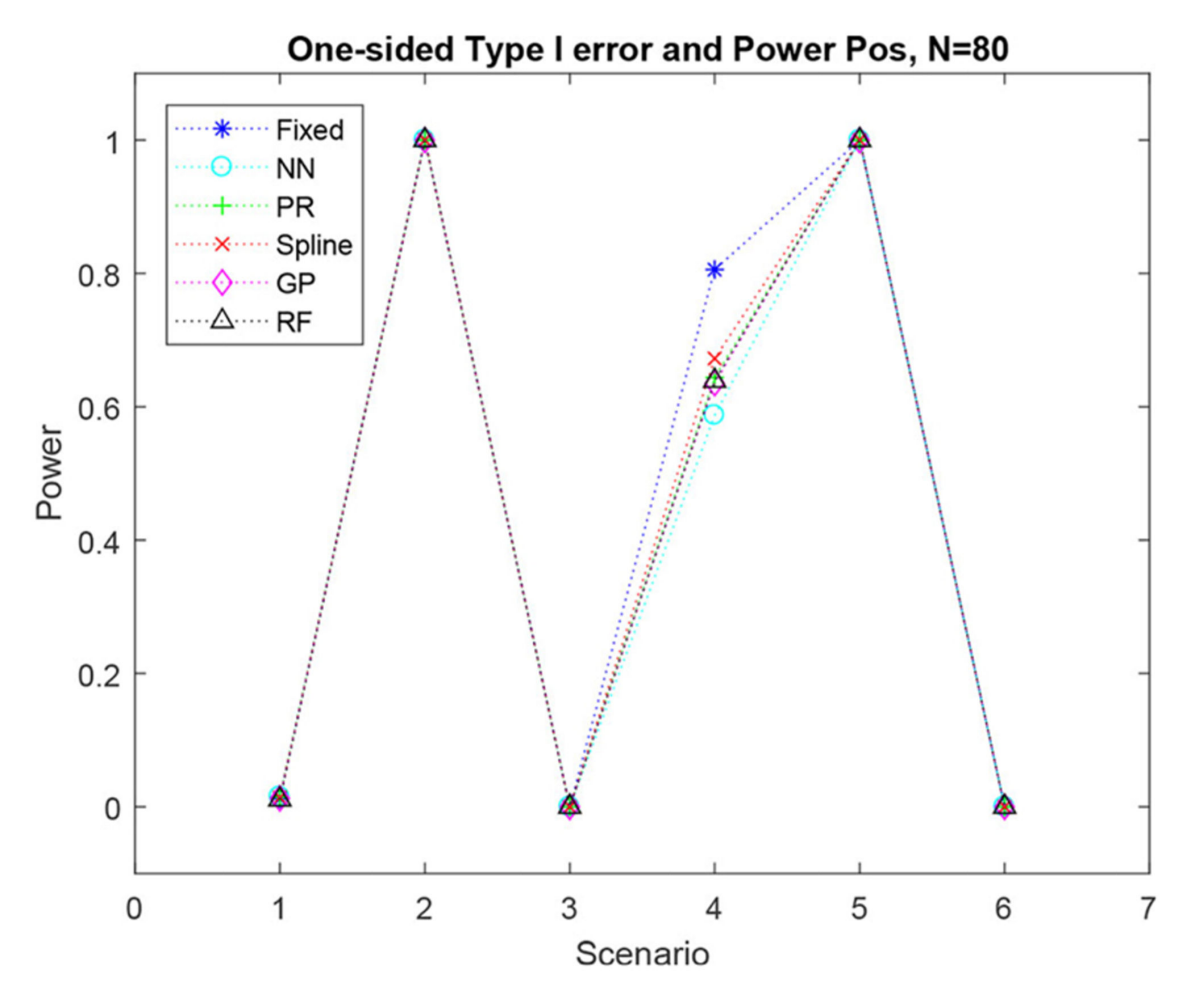
Simulated one-sided type I error and power for biomarkers *x_n_* ≥ 0 for six scenarios with a sample size of *N* = 80.

The one-sided power produced for biomarker positive patients was slightly higher for FR than the regression methods for scenario four. However, the power produced by the regression methods was very similar to that produced by FR for all other scenarios. This can be seen in Figure 4. The one-sided power produced in patients who are biomarker negative, is shown in the supplementary material.

#### 4.3.3 Two-sided type I error and power

**Figure 5:**
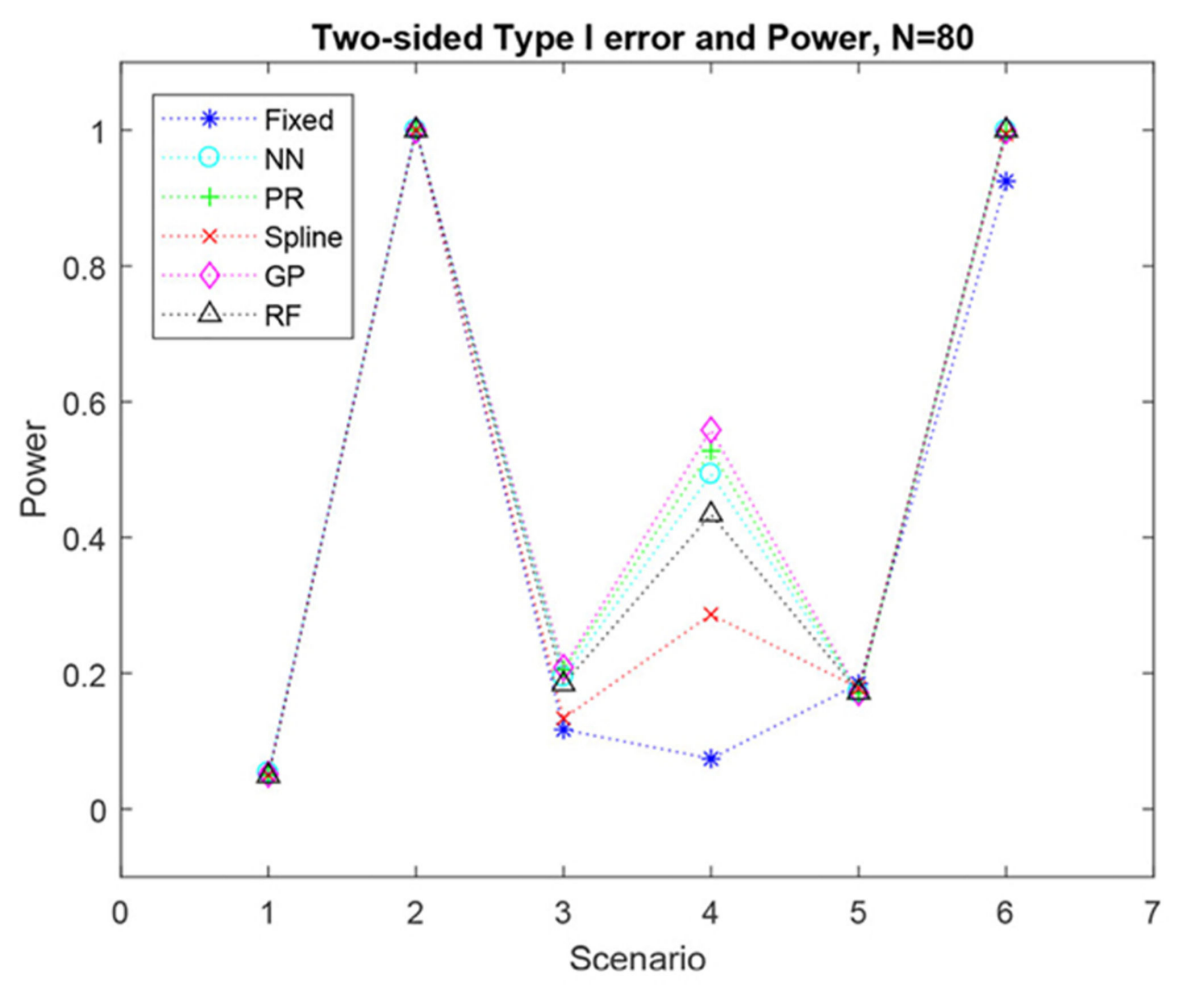
Simulated two-sided type I error and overall power, for six scenarios with a sample size of *N* = 80.

When we investigate the overall two-sided type I error and power, Figure 5 shows more of a difference between the methods. It shows FR and the regression methods produce a similar power for scenarios two and five. However, in scenarios three, four and six, the regression methods all produce a higher power than the FR method. For these scenarios the regression methods that produce the largest power are Gaussian processes, polynomial regression and the nearest neighbor method.

**Figure 6:**
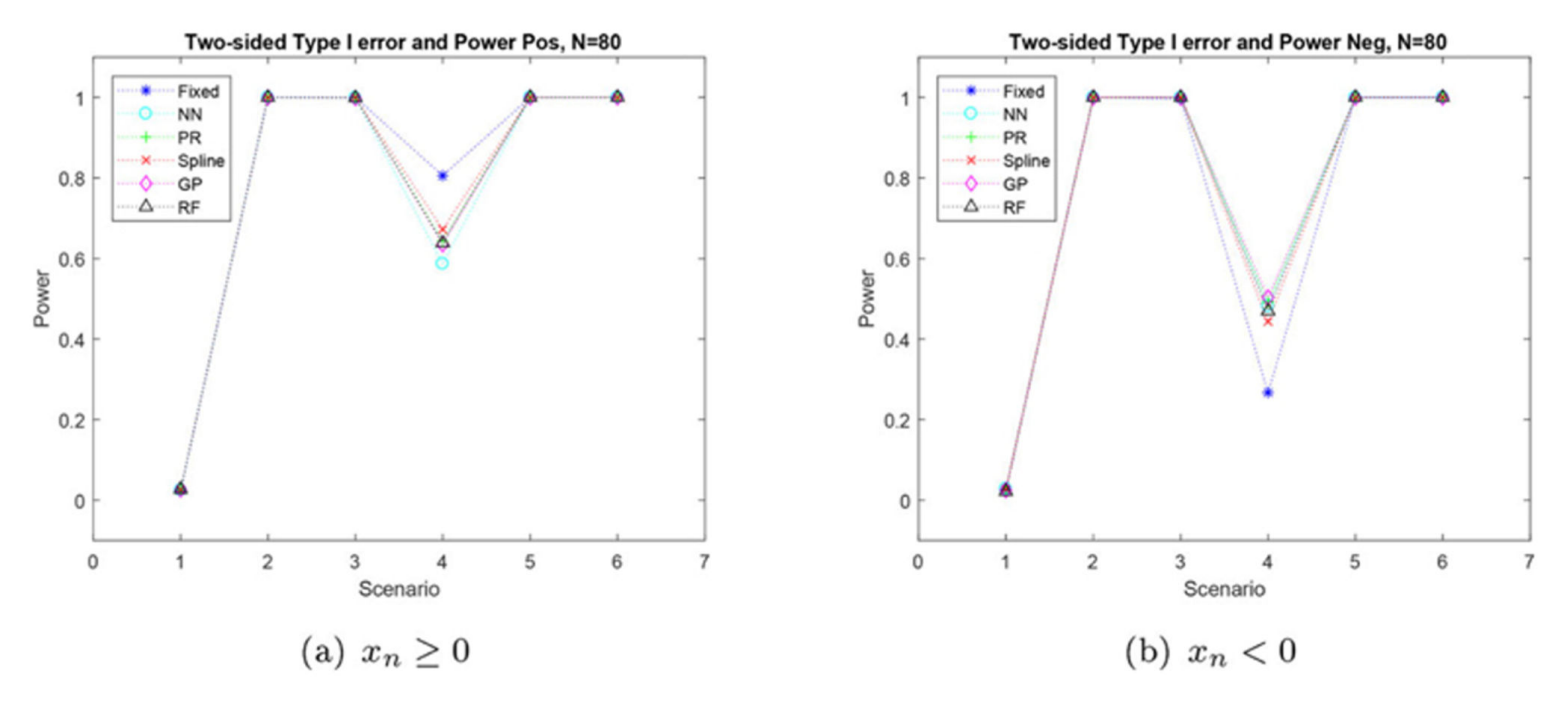
Simulated two-sided type I error and power for biomarkers *x_n_* ≥ 0 (a) and biomarkers *x_n_* < 0 (b), for six scenarios with a sample size of *N* = 80.

If we only take into account patients who are biomarker positive, Figure 6a shows a dip in power for scenario four, when *N* = 80. This is due to scenario four having the smallest difference between the two treatments for biomarker positive patients. The power produced by FR is 0.2 larger than the power produced by the regression methods, for this scenario.

Figure 6b shows the power of the trial when the sample size is *N* = 80 for only biomarker negative patients, where the FR method produces the smallest power for scenario four. As discussed previously this is due to the regression methods assigning more people to their better treatments and hence, producing a larger difference in the mean outcome of the two treatments.

We include more plots for this simulation study for varying trial size, *N*, in the supplementary materials.

## 5 Case study simulation

To highlight the versatility of the proposed method, we now also illustrate its utility on the basis of our motivating example described in Sec. 2. Here, the outcome is time to death and we investigate two binary biomarkers. Four different scenarios are considered and, as in the simulation above, a simulation size of 10 000 is used for all regression methods except for splines and Gaussian processes, where 1 000 runs are used due to the computational burden.

The following simulations, focus on a trial size of *N* = 233 as in the study (NCT00836654) and we further investigate the case study using sample sizes *n* = 40 and *N* = 80 to determine how a smaller trial size would affect the proposed method. We will compare the average outcome of the two treatments, the control *k* = 0 and catumaxomab *k* = 1, using the logrank test to find the twosided power for the different regression methods.

We assume that the two biomarkers are binary, such that if a patient’s *RLC* ≤ 13%, their first biomarker value is *x*_*n*, 1_ = 1 and if their *RLC* > 13%, their first biomarker value is *x*_*n*, 1_ = 2. If a patient’s *KI* < 70%, their second biomarker value is *x*_*n*, 2_ = 1 and if their *KI* ≥ 70%, their second biomarker value is *x*_*n*, 2_ = 2. We assume that the two biomarkers are independent and, using the results from Heiss et al. (2014), the probability of a patient’s *RLC* > 13% = 0.6824, and the probability of a patient’s *KI* ≥ 70% = 0.8584.

Here, the outcome variable is overall survival (OS), thus, our assumption of knowing the outcome of patient *n* before patient *n* + 1 arrives no longer holds. Hence, we incorporate censored data into our simulation and the regression methods are adjusted to handle censored data. We include censoring due to drop out and not knowing the survival time of patient *n* before patient *n* +1 arrives into the trial. The specifications for each regression method are described in Appendix D. We do not investigate the random forest regression method here, due to us predicting the outcome of patients based on two binary biomarkers. This restricts the size and possible variety of the trees produced.

The study lasted roughly 1250 days (Heiss et al. 2014), it included 233 patients, so on average the patients arrived every $\frac{1250}{233}=5.36\;\approx 5\text{ days}$. In the simulation, we assume the time between each patient’s arrival time is taken from the Poisson distribution with mean five. After the last patient is allocated a treatment, each patient is followed up for an extra six months. At this time if their death has not been recorded they are assumed censored. We also included censoring due to drop out in the simulation. The rate at which patients drop out of the trial varies between scenarios and is detailed below. If patient *n* was assigned to be censored, the censored time was chosen from a uniform distribution between 0 and their previously assigned OS outcome. The censored and OS times are integer values, to represent in application deaths are normally recorded per day, rather than per hour or minute.

### 5.1 Scenarios

The four scenarios we investigate are:

1. No treatment effect for all patients. Neither RLC nor KI are significant biomarkers. We simulate the outcome of all patients from an exponential distribution with mean 98 days for both treatments, as the median OS for the control treatment is reported as 68 days by Heiss et al. (2014). 20% of the patients assigned to the control treatment and 20% of the patients assigned to the catumaxomab treatment were censored due to drop out.
2. No treatment effect for all patients. We simulate the outcome of all patients from an exponential distribution with mean 98 days for both treatments, as the median OS for the control treatment is reported as 68 days by Heiss et al. (2014). 20% of patients assigned to the control treatment and 8% of the patients assigned to the catumaxomab treatment were censored due to drop out. This was estimated from the reported censoring rates and hazard ratios (HR) (Heiss et al. 2014).
3. Treatment increases OS for all patients, where the *RLC* is a predictive biomarker. The *KI* is not a predictive biomarker. We simulate the outcomes of the control treatment from an exponential distribution with mean 98 days. However, catumaxomab OS times are generated using an exponential distribution with mean 141 days when a patient has *RLC* ≤ 13% and with mean 189 days when a patient has *RLC* > 13%. These means are calculated using the reported hazard ratios (HR = 0.695 for *RLC* ≤ 13% and HR = 0.518 for *RLC* > 13%) (Heiss et al. 2014). 20% of patients assigned to the control treatment and 8% of the patients assigned to the catumaxomab treatment were censored due to drop out.
4. Both the *RLC* and *KI* are predictive biomarkers. We simulate outcomes from the control treatment from an exponential distribution with mean 98 days. Whereas, the experimental treatment gives outcomes from an exponential distribution with mean 90 days when a patient has *RLC* ≤ 13% and *KI* < 70, with mean 160 days when a patient has *RLC* ≤ 13% and *KI* ≥ 70, with mean 170 days when a patient has *RLC* > 13% and *KI* ≥ 70 and with mean 200 days when a patient has *RLC* > 13% and *KI* ≥ 70. These means are based on the reported hazard ratios (HR = 0.695 for *RLC* ≤ 13%, HR = 0.518 for *RLC* > 13%, HR = 0.567 for *KI* > 70% and HR = 0.582 for overall treatment effect), shown by Heiss et al. (2014). 20% of patients assigned to the control treatment and 8% of the patients assigned to the catumaxomab treatment were censored due to drop out.

### 5.2 Results

**Figure 7:**
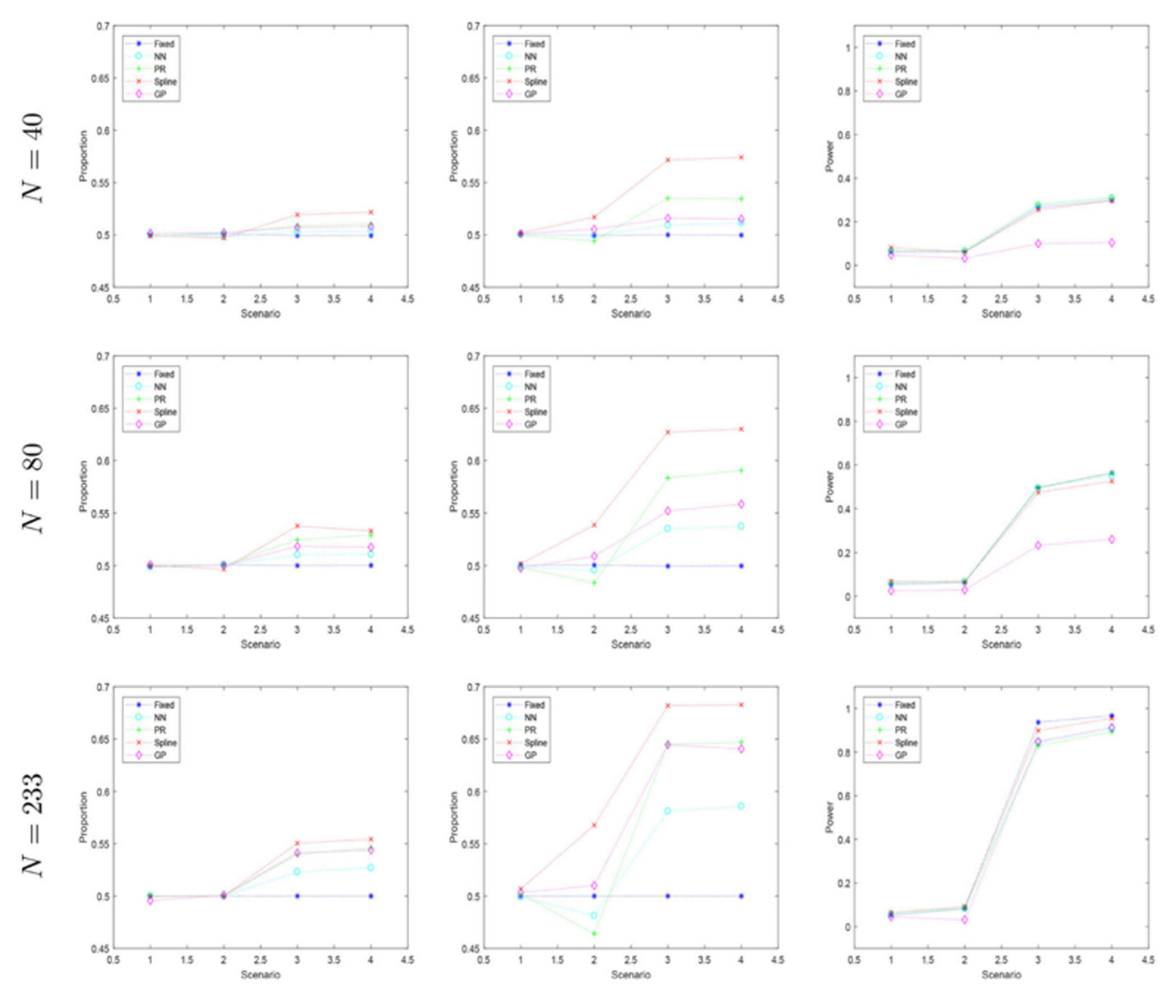
Simulated proportion of patients on their best treatment, on catumaxomab and type I error and power of the trial for four scenarios with a sample size of *N* = 40, 80 & 233.

Figure 7 displays the three different performance characteristics, for our three different sample sizes. Here, the top row shows the results of our simulation when the sample size is *N* = 40, the second row displays the results when the sample size is *N* = 80 and the bottom row has a sample size of *N* = 233. The first column displays the proportion of patients given their best treatment, the second column shows the proportion of patients allocated to catumaxomab and the final column indicates the type I error and power of each scenario for each of the three sample sizes investigated.

The first column in Figure 7 shows all the regression methods produce a higher proportion of patients assigned to their best treatment than the equal fixed randomization method, and each regression method tends to assign more patients to their best treatment as the sample size increases. The proportion of patients on their best treatment is at most 0.554 when using the spline regression method and the sample size is *N* = 233. The methods which perform best are splines, Gaussian processes and polynomial regression. Interestingly, Gaussian processes and polynomial regression also performed best in the simpler scenarios investigated in Sec. 4 and hence, are a robust choice to use.

The second column in Figure 7 indicates the maximum proportion of patients on catumaxomab to be 68.2% for scenarios three and four, when the sample size is *N* = 233. Catumaxomab is on average the best treatment for all patients in scenario three and for three out of four subgroups of the trial population in scenario four. However, when we look at the first column, less than 55% of patients are assigned their best treatment by splines in scenario three and four. This difference is caused by the variation within the data produced. Even though, in scenario three catumaxomab was the best treatment on average, due to simulating the patient’s outcomes from exponential distributions, sometimes the control treatment was actually better for individual patients. This can be thought of as the patients possessing other biomarkers (which we have not accounted for in the study) which cause them to produce a better outcome on the control treatment.

Even though scenario two has treatments which produce the same outcomes on average, most regression methods do not assign patients to both treatments equally. Splines assign many more patients to catumaxomab in scenario two than in scenario one. Whereas, polynomial regression assign fewer patients to catumaxomab in scenario two than in scenario one. This difference is due to the different censoring rates for each treatment (20% on the control and 8% on catumaxumab) in scenario two. As the sample size increases, the more unbalanced the treatment allocation becomes for all regression methods. However, splines produce the value furthest from 50% and Gaussian processes produce the value closest to 50%.

The bottom right plot of Figure 7 indicates all methods result in large power in scenarios three and four, when the sample size is large. The smallest power, produced by polynomial regression, is still above 0.8. Traditionally, trial designs should have a theoretical minimum power of 0.8 for them to be considered a feasible design for an actual clinical trial. Hence, all methods produce a large enough power to be a feasible trial design when the sample size is *N* = 233. As the sample size decreases, the power of all regression methods decrease. Gaussian processes in particular, lose the most power.

One challenge of CARA designs is the selection of biomarkers, i.e., including extra biomarkers that are non-informative. In the four scenarios above, both biomarkers, RLC and KI, were assumed to be predictive and were used to assign patients to their best treatment. This assumption was met in Scenario four where both RLC and KI were truly predictive. In Scenario three, however, only RLC was predictive and including KI in the model to allocate patients was unnecessary. When comparing the results of these two scenarios, however, we find that there is not a large difference in patient benefit or power. This suggests our method still performs well even if non-informative biomarkers are included.

## 6 Conclusions and further work

Thus far, RAR designs have not been used often in clinical trials, due to their lack of ability to produce a high power. However, rare diseases appear to be the most promising application area where RAR designs can be used. In this work we have introduced a personalized RAR approach that can be utilized with a large range of outcome types (including binary, categorical, continuous or survival) and biomarker types (including binary, categorical and continuous).

A key component of the approach is the regression methods used. We found that Gaussian processes performed well for all situations investigated. It produced the best performance in the simulation in Sec. 4, when a single continuous biomarker was used. Although, it did require a larger sample size to perform well in more complex settings (shown in Sec. 5, when two binary biomarkers were used to predict a survival outcome). In the more complex setting, when the sample size was small, polynomial regression performed better. Polynomial regression also performed well when a single continuous biomarker was used. Therefore, we recommend Gaussian processes as the regression method of choice in simple situations or when the sample size is large, otherwise we recommend polynomial regression.

A key challenge of CARA designs is the selection of informative biomarkers. While there is some suggestion that our method still performs well when non-informative biomarkers are included, a more parismonious approach might be preferred due, for example, to cost or invasiveness of measurement. The main challenge is that control of error rates is difficult when selection is based on the same data. Therefore, either two-stage procedures (similar to that suggested by Freidlin and Simon (2005)) are used or an exploratory framework (i.e., without strict error control), as in Chen et al. (2012), is utilized (see also the review by Ondra et al. (2016)).

Besides the regression approach used, the proposed RAR design depends on the chosen sequence, *π_n_*, which is a linear decrease from $\pi_{10}=\frac{1}{K}$ to *π_N_* = 0.1. Future investigations will explore other sequences of *π_n_*, such as an exponentially decreasing sequence, to evaluate if we can assign more patients to their best treatment without decreasing the power of the design.

The method could be extended to include biomarkers of different types. For example, we could include several continuous, categorical and binary biomarkers with complex non-parametric relationships with the outcome variable, and explore how this would effect the method. However, even though each regression method explored in the above simulations has the potential to model more complex relationships, this is not necessarily useful in practice. In application the number of known markers for a disease will be small. Therefore, if the method were to be used in a clinical trial, only a small number of biomarkers would be included in the prediction models.

Finally, in all scenarios investigated, we consider a continuous outcome and a survival outcome. This method could be extended to include a surrogate endpoint. A surrogate endpoint is defined by Aronson (2005), as ‘a biomarker intended to substitute for a clinical endpoint’. These surrogate endpoints are used, because they are more practical to measure. They occur earlier in time than the actual primary endpoint, and they give you an idea of what the primary outcome will actually be in that patient.

## Supplementary Material

Supplementary File

Appendix A
