# Supplementary material for: Using biomarkers to allocate patients in a response-adaptive clinical trial
Source: Commun Stat Simul Comput. Author manuscript; Available in PMC 2023 Dec 2. (PMC7615340; doi:10.1080/03610918.2021.2004420)
Supplement: Supplementary File [file EMS144030-supplement-Supplementary_File.pdf]

Supplementary Material for 'Using biomarkers to allocate patients in a response-adaptive clinical trial'

1 Additional Simulation Results

Below, we show the results of our simulations as described in the main paper for $N = 40, 80,$ and 120.

1.1 Ethical Measure

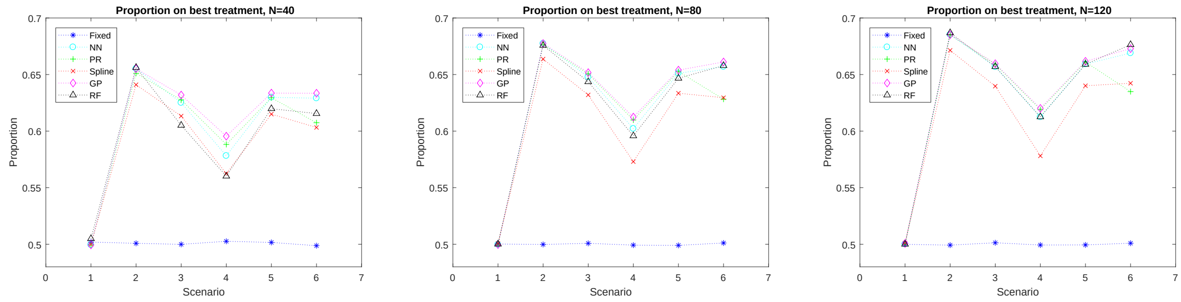

(a) $N = 40$ (b) $N = 80$ (c) $N = 120$

Figure 1: Simulated proportion of patients on their best treatment, when the trial size is $N = 40$ (a), $N = 80$ (b) and $N = 120$ (c) for 6 scenarios.

1.2 Type I error and Power

1.2.1 One-sided:

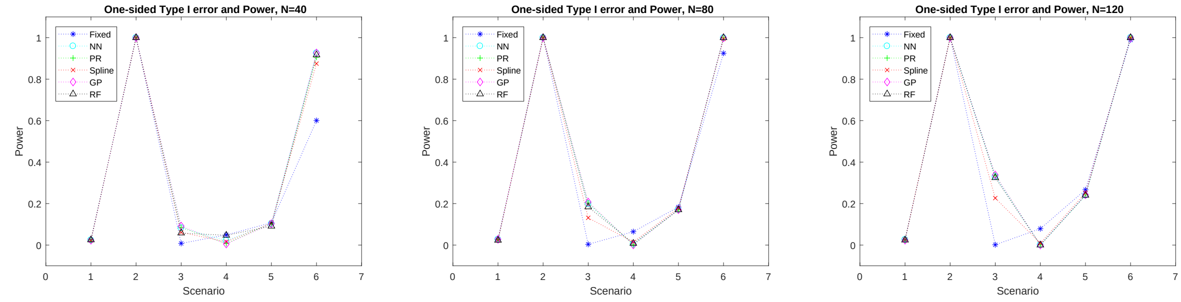

(a) $N = 40$ (b) $N = 80$ (c) $N = 120$

Figure 2: Simulated one-sided type I error and overall power, when the trial size is $N = 40$ (a), $N = 80$ (b) and $N = 120$ (c) for 6 scenarios.

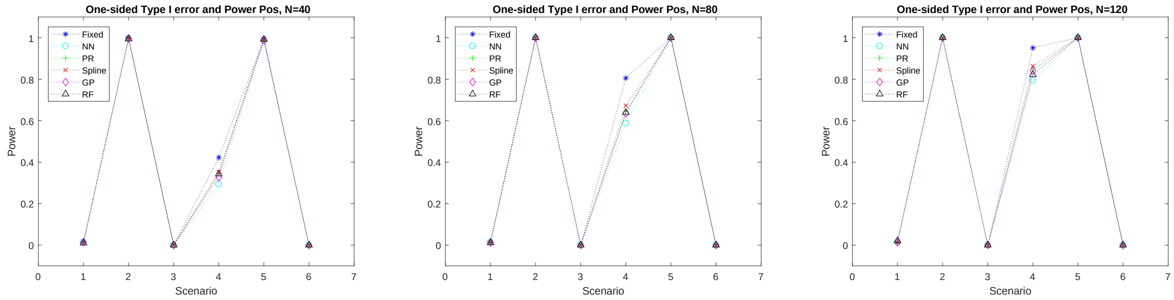

(a) $N = 40$ (b) $N = 80$ (c) $N = 120$

Figure 3: Simulated one-sided type I error and power for biomarkers $x_n \geq 0$, when the trial size is $N = 40$ (a), $N = 80$ (b) and $N = 120$ (c) for 6 scenarios.

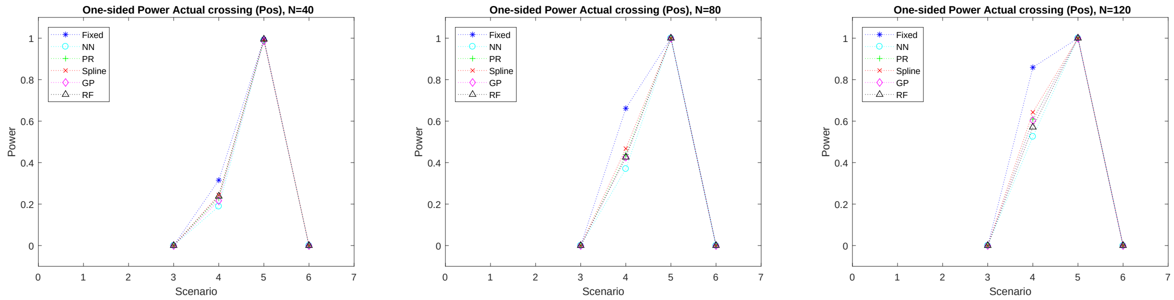

(a) $N = 40$ (b) $N = 80$ (c) $N = 120$

Figure 4: Simulated one-sided power for biomarkers $x_n \geq X$, when the trial size is $N = 40$ (a), $N = 80$ (b) and $N = 120$ (c) for 6 scenarios.

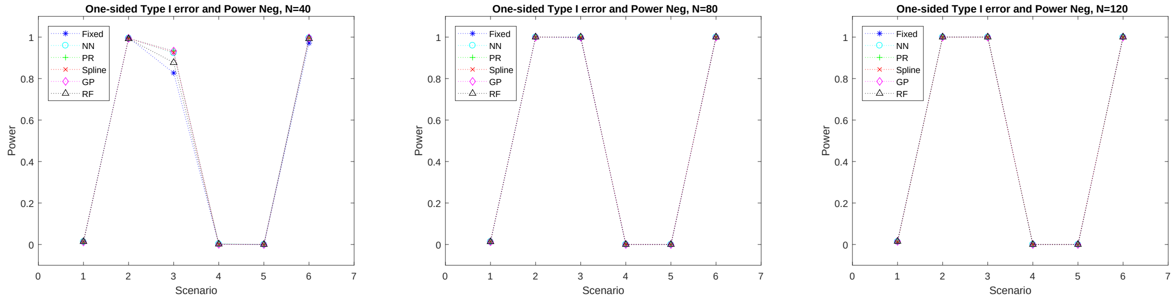

(a) $N = 40$ (b) $N = 80$ (c) $N = 120$

Figure 5: Simulated one-sided type I error and power for biomarkers $x_n < 0$, when the trial size is $N = 40$ (a), $N = 80$ (b) and $N = 120$ (c) for 6 scenarios.

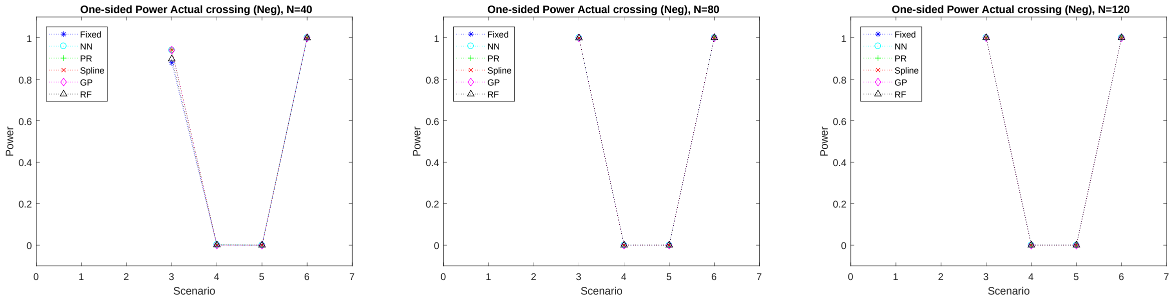

(a) $N = 40$ (b) $N = 80$ (c) $N = 120$

Figure 6: Simulated one-sided power for biomarkers $x_n < X$, when the trial size is $N = 40$ (a), $N = 80$ (b) and $N = 120$ (c) for 6 scenarios.

1.2.2 Two-sided:

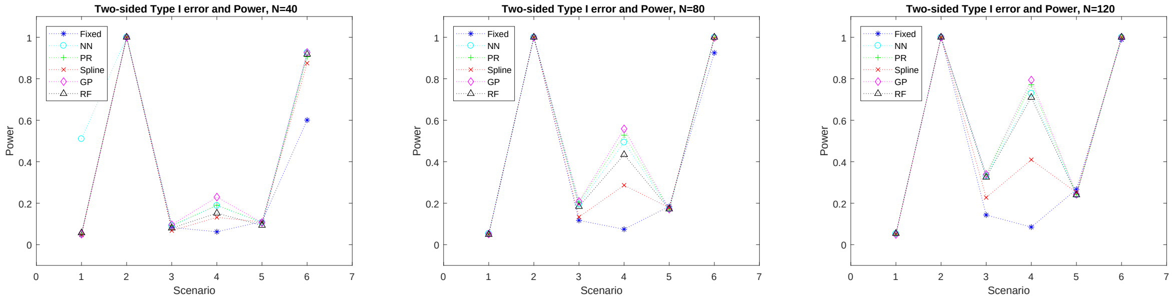

(a) $N = 40$ (b) $N = 80$ (c) $N = 120$

Figure 7: Simulated two-sided type I error and overall power, when the trial size is $N = 40$ (a), $N = 80$ (b) and $N = 120$ (c) for 6 scenarios.

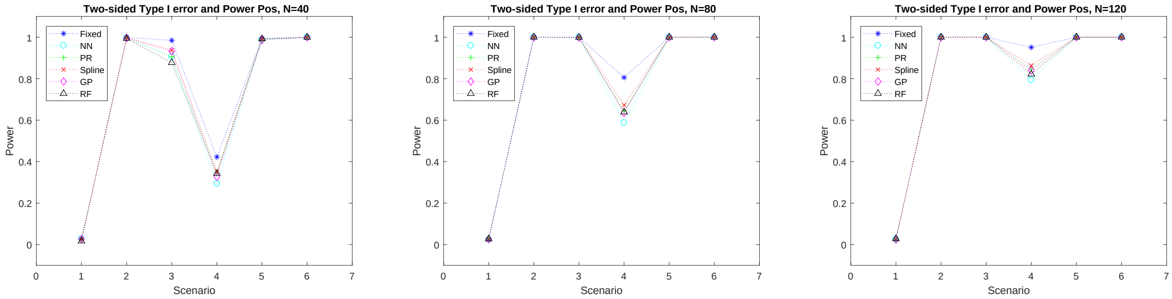

(a) $N = 40$ (b) $N = 80$ (c) $N = 120$

Figure 8: Simulated two-sided type I error and power for biomarkers $x_n \geq 0$, when the trial size is $N = 40$ (a), $N = 80$ (b) and $N = 120$ (c) for 6 scenarios.

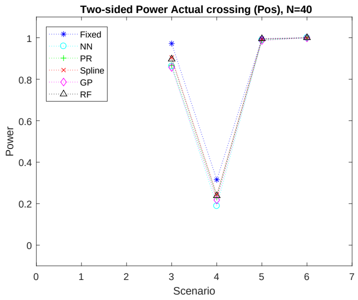

(a) $N = 40$

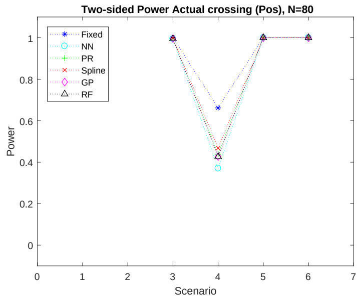

(b) $N = 80$

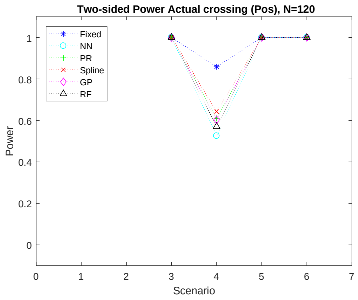

(c) $N = 120$

Figure 9: Simulated two-sided power for biomarkers $x_n \geq X$, when the trial size is $N = 40$ (a), $N = 80$ (b) and $N = 120$ (c) for 6 scenarios.

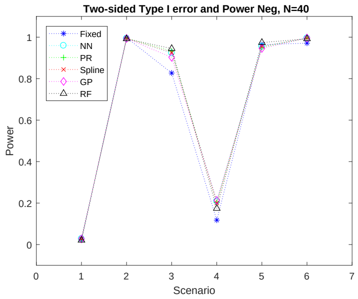

(a) $N = 40$

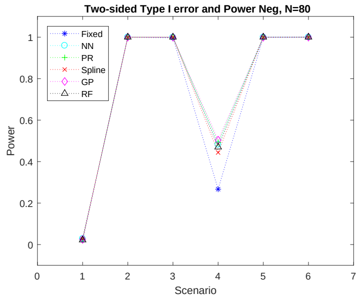

(b) $N = 80$

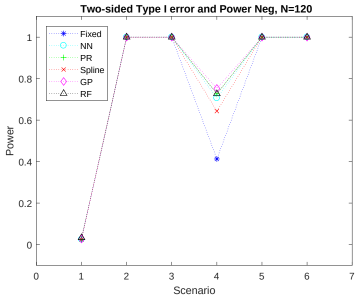

(c) $N = 120$

Figure 10: Simulated two-sided type I error and power for biomarkers $x_n < 0$, when the trial size is $N = 40$ (a), $N = 80$ (b) and $N = 120$ (c) for 6 scenarios.

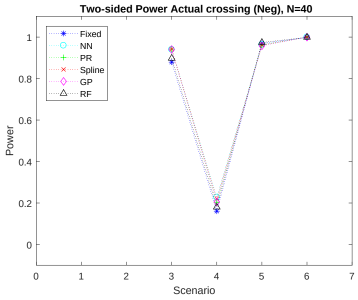

(a) $N = 40$

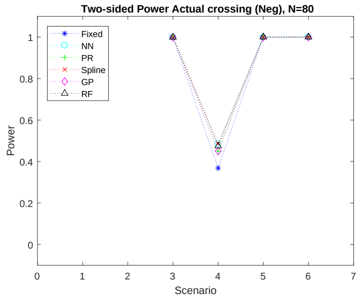

(b) $N = 80$

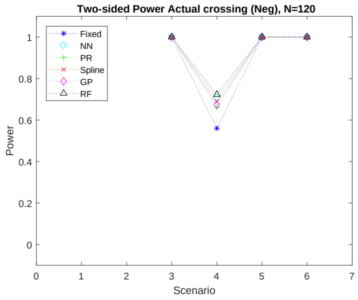

(c) $N = 120$

Figure 11: Simulated two-sided power for biomarkers $x_n < X$, when the trial size is $N = 40$ (a), $N = 80$ (b) and $N = 120$ (c) for 6 scenarios.
