# Supplementary material for: Using biomarkers to allocate patients in a response-adaptive clinical trial
Source: Commun Stat Simul Comput. Author manuscript; Available in PMC 2023 Dec 2. (PMC7615340; doi:10.1080/03610918.2021.2004420)
Supplement: Appendix A [file EMS144030-supplement-Appendix_A.pdf]

Appendix A. Regression procedures

A large number of potential regression procedures can be used to estimate the functions $f_k(x) \forall k \in \{1, K\}$. In this work we will explore a selection of these which are subsequently described. Consider our patient outcome model, $Y_{n,k} = f_k(x_n)$. All of the following regression procedures can be used when the biomarker values $x_1, ..., x_N$ are continuous or when they are binary or categorical.

A.1. $Z_{N_{k,n}}$ nearest neighbor

Let $d(x_1, x_2)$ be a distance measure between the biomarkers of patients one and two, x_1 and x_2. This distance measure is chosen based on the number of biomarkers being investigated and their type. Let $J_k(n)$ be the set of $Z_{N_{k,n}}$ patients who have been allocated to treatment k and are closest to patient n as defined by the distance measure, d (Yang and Zhu 2002). Here, $N_{k,n}$ is the number of patients in the trial given treatment k, when patient n enters the trial.

For each treatment k calculate the mean of the observed outcomes for the $Z_{N_{k,n}}$ closest neighboring points to x_n (most similar patients to patient n), $\hat{f}_{k,y_{n-1,k}}(x_n) = \frac{1}{Z_{N_{k,n}}} \sum_{j \in J_k(n)} y_{j,k}$. If the biomarker values are categorical, they are transformed into dummy variables in order for the regression method to work.

The tuning parameters for this method are the distance measure, $d(x_1, x_2)$ and the number of neighbors, $Z_{N_{k,n}}$. The more nearest neighbors are used, the smoother the estimate $\hat{f}_{k,y_{n-1,k}}(x_n)$ is. However, the estimate will struggle to detect small changes and it will not be as accurate in the tails. If too few nearest neighbors are used, the estimate $\hat{f}_{k,y_{n-1,k}}(x_n)$ will not be smooth, as it will react to small changes. The number of nearest neighbors used should vary depending on how many patients $N_{k,n}$ have been assigned to treatment k, when patient n enters the trial. The more patients on treatment arm k the more information we have and the more nearest neighbors can be used.

A.2. Polynomial regression

These models fit an mth order polynomial relationship between independent variable(s) and a dependent variable described by Montgomery, Peck, and Vining (2012) in equation $f_k(x_n) = \beta_0 + \beta_1 x_n + \beta_2 x_n^2 + \cdots \beta_m x_n^m + \epsilon_n$, where ϵ_n is the error term, which is assumed to be normally distributed with a mean of 0 and finite variance. If the biomarker values are categorical, they are transformed into dummy variables in order for the regression method to work.

The tuning parameter for this procedure is the order of the polynomial we fit to the data. The higher the degree of the polynomial, the more likely it is to over-fit to the data and the estimate $\hat{f}_{k,y_{n-1,k}}(x_n)$ will not be smooth as higher orders will start to take into account the random error term. At the same time, if the degree of the polynomial is too small, $\hat{f}_{k,y_{n-1,k}}(x_n)$ will under-fit the data and the regression line will be smooth, but will not detect the small changes.

A.3. Spline regression

Spline Regression is described as piecewise polynomial regression, by Huang (2003). The data is split into $S+1$ subsets (Friedman 1991), and a polynomial function is fitted to each subset. These polynomial functions can be of

different orders but they must be constrained such that they are continuous where the subsets of the data meet, as stated by Durrleman and Simon (1989).

The entire interval of biomarker values x are split into $S+1$ separate subsets by 'knots', hence, $S+1$ polynomial functions are estimated. We label the knots as $x_s^* \forall s \in \{1, S\}$. The polynomials $g_s \forall s \in \{1, S+1\}$ are then fitted together into one continuous curve $\hat{f}_{k,\boldsymbol{y}_{n-1,k}}(x_n)$. Such that when a patient's biomarker is smaller than the first knot, $x_n \leq x_1^*$ then $\hat{f}_{k,\boldsymbol{y}_{n-1,k}}(x_n) = g_1(x_n)$ and when a patient's biomarker lies between the first and second knots, $x_1^* \leq x_n \leq x_2^*$ then $\hat{f}_{k,\boldsymbol{y}_{n-1,k}}(x_n) = g_2(x_n)$, e.t.c. Thus, at each knot $s \in \{1, S\}$, where polynomial functions g_s and g_{s+1} meet, the value $g_s(x_s^*)$ must be equal to $g_{s+1}(x_s^*)$ and the first $m-1$ derivatives of $\hat{f}_{k,\boldsymbol{y}_{n-1,k}}(x_n)$ (where m is the order of the polynomials g_s) must be continuous (Friedman 1991).

The three tuning parameters for this method are the number of knots and their positions and the degree of the polynomial which is fitted between each pair of knots.

A.4. Gaussian processes

Gaussian Processes are described by Williams and Rasmussen (2006) as a generalization of the Gaussian probability distribution. Multiple functions are drawn at random from the prior distribution specified by a particular Gaussian process. This prior distribution represents our beliefs about the function $f_k(x)$, which we will observe. This Gaussian process prior is combined with a Gaussian likelihood to calculate a posterior Gaussian process.

As patients enter the trial and are given treatment k, we collect their data $(x_n, y_{n,k})$ and only consider sample functions which pass through these data points. Gaussian processes calculate the mean values of *all* these sample functions. As more patients are given treatment k, more data points can be used to estimate the function $f_k(x)$ and hence, the number of sample functions which pass through these data points will decrease.

Choosing the prior distribution can reduce the number of possible sample functions that are considered. Other characteristics of the function $f_k(x)$ such as smoothness and it's stationarity can also be controlled via the covariance function in order to reduce the number of possible sample functions. Here a covariance function $c(x, x')$ describes the relationship between two points $(x, f_k(x))$ and $(x', f_k(x'))$, as stated by Schulz, Speekenbrink and Krause (2016). In most situations, we assume that, when the distance between two points x and x' is small, the two points are closely correlated, whereas, when the distance between the two points is large, they are not closely correlated. The covariance function must represent this.

We collect data points $(x_i, y_{i,k})$ for $i \in \{1, N_{k,n}\}$, where $N_{k,n}$ is the number of patients in the trial given treatment k, when patient n enters the trial. The covariance function, c, is found for all combinations of these $N_{k,n}$ data points and stored in the matirx C (Ebden 2008).

When the next patient with biomarker x_n arrives, the covariance function between x_n and all $N_{k,n}$ data points already collected is found $C^* = [c(x_n, x_1), \cdots, c(x_n, x_{N_{k,n}})]$, and between x_n and itself, $C^{**} = c(x_n, x_n)$.

The joint multivariate Gaussian distribution of the vector of observed outcomes, $\boldsymbol{y}_{n-1,k}$ and the estimated function is then shown by Ebden (2008) to be,

$$\begin{bmatrix} \boldsymbol{y}_{n-1,k} \\ \hat{f}_k(x_n) \end{bmatrix} \sim N\left(\mathbf{0}, \begin{bmatrix} C & C^{*T} \\ C^* & C^{**} \end{bmatrix}\right).$$

From this we find the conditional estimate of the outcome for patient n, given the data from previous patients, $\hat{f}_{k,\boldsymbol{y}_{n-1,k}}(x_n) \equiv \hat{f}_k(x_n) \mid \boldsymbol{y}_{n-1,k}$, for all treatments $k \in \{1, K\}$ as, $\hat{f}_k(x_n) \mid \boldsymbol{y}_{n-1,k} \sim N(C^* C^{-1} \boldsymbol{y}_{n-1,k}, C^{**} - C^* C^{-1} C^{*T})$. If the biomarker values are categorical, they are transformed into dummy variables in order for the regression method to work.

A.5. Random forests

Random Forests are the aggregate of a finite number of regression trees.

A regression tree is a method to create a set of rules on independent variable(s), in order to partition the data into separate subgroups. These subgroups should contain a dependent variable of similar value to each other but different to the value of the dependent variable of other subgroups. Segal (1988) explains the regression tree chooses the best independent variable to introduce a rule on, using goodness-of-split criterion, in order to split the data into consecutively smaller groups. Each rule focuses on only one independent variable and each rule has a binary outcome, as stated by Prasad, Iverson and Liaw (2006). This is seen in Figure A1, where each rule has a binary

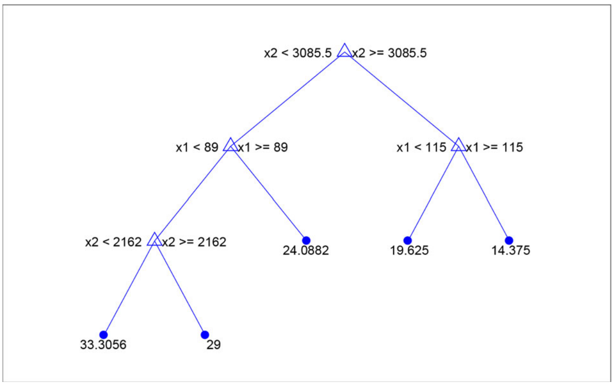

Figure A1. Example Regression Tree.

outcome, e.g., $x1 < 89$ or $x1 \geq 89$ and produces two subgroups. This splitting procedure is repeated until a termination criterion is met, at which point the resulting subgroup of the data (called a terminal node) will not be split further. The number of ways in which a variable can produce a split depends on the type of variable.

Termination criteria include: have a maximum number of outcomes in each terminal node or have a minimum improvement in the least squares criterion, resulting from the best split. However, if these thresholds are too small then overfitting can occur, but if these thresholds are too large, underfitting could occur (Segal 1988).

Random forests can be used to combat the issues of underfitting and overfitting in regression trees, as stated by Prasad, Iverson and Liaw (2006). Instead of using the data to create just one regression tree, in the random forest method bootstrap samples are drawn from the data to construct multiple trees. Each bootstrapped sample produces a regression tree, however, each 'best' split in a tree is chosen from a randomized subset of all independent variables. The trees are grown to maximum size and then averaged. It is recommended by Oshiro, Santoro and Baranauskas (2012) to use between 64 and 128 trees in a random forest. The number of trees in a forest is the tuning parameter for this method.

Appendix B. Simulation scenarios

The following table displays the underlying functions between a patient's biomarker and the outcome produced in that patient if given each treatment, in the simulation from Sec. 4.

Table B1. Simulation Scenario Summary.

Scenario	Treatment one	Treatment two
One	0	0
Two	$20(\frac{1}{\exp(0.002x)+1})-10$	$20(\frac{1}{\exp(0.002x)+1})-4$
Three	0	$20(\frac{1}{\exp(0.02(x+8))+1})-10$
Four	$20(\frac{1}{\exp(0.02(x+5.2))+1})-10$	$20(\frac{1}{\exp(0.011x)+1})-10$
Five	$20(\frac{1}{\exp(0.01(x+16))+1})-10$	$20(\frac{1}{\exp(-0.01x)+1})-10$
Six	-5	8 for $x_n < -8$<br>-8 for $x_n \geq -8$

Appendix C. Simulation specifications for regression methods

In the nearest neighbor method, we use the Euclidean distance to measure the similarity between patients. We also use $Z_{N_{k,n}} = 3$ neighbors when the number of patients given treatment k, when patient n arrives into the trial is $N_{k,n} \leq 20$. As there are only a small number of patients in the trial at this time using 3 neighbors will still allow a moderately good estimate, $\hat{f}_{k,y_{n-1,k}}(x_n)$ to be calculated. We use $Z_{N_{k,n}} = 4$ when the number of patients given treatment k, when patient n arrives into the trial is $20 < N_{k,n} \leq 40$ and we use $Z_{N_{k,n}} = 6$ when the number of patients given treatment k, when patient n arrives into the trial is $N_{k,n} > 40$. This keeps the estimate $\hat{f}_{k,y_{n-1,k}}(x_n)$ smooth when we have a large number of patients in the trial. We used leave-one-out cross-validation to select how many neighbors we would use for our simulation. However, in a different application the best number of nearest neighbors could change.

For polynomial regression, we use the function ‘polyfit’ (Matlab polyfit 2016) in matlab with a polynomial of degree 3 for practical reasons. In application, the relationship between a biomarker and the effectiveness of a treatment will not normally be of a degree above a cubic. However, it is still of a high enough degree that it can track a non-parametric relationship.

In our method we use the interpolating cubic spline function ‘csapi’ (Matlab csapi 2016) in matlab, where each polynomial g is of order 3. For interpolating splines the mth (3rd) derivative of the function $\hat{f}_{k,y_{n-1,k}}(x_n)$ must be continuous at the first and last knots. We choose $S = 3$ knots. The first knot is placed at the smallest recorded biomarker value for each treatment k, when patient n enters the trial. The second knot is placed at the biomarker which is $1 + \frac{N_{k,n}}{3}$ (rounded up) next largest and the third knot is placed at the biomarker which is $1 + \frac{2N_{k,n}}{3}$ (rounded up) next largest. For our scenarios, $S = 3$ knots is best, however, this may not be the case for other scenarios.

We use the Gaussian process function ‘fitrgp’ (Matlab fitrgp 2016) in matlab, using the default settings in our Gaussian processes.

We use the random forest function ‘TreeBagger’ (Matlab treebagger 2016) in matlab with 100 aggregated regression trees, as it seems appropriate from the literature.

Appendix D. Case study simulation specifications for regression methods

The nearest neighbor method is adapted to take account of the two binary biomarkers and the censored data. We use the Euclidean distance to measure the similarity between patients. If two patients are an equal distance from a third patient, the one with the least common combination of biomarkers is taken to be ‘closer’. Here we used cross validation to find the number of nearest neighbors which produces the most patients on their best treatment. We used $Z_{N_{k,n}} = 3$ neighbors when there were 25 patients or less assigned to a treatment k, when patient n enters the trial ($N_{k,n} \leq 25$), $Z_{N_{k,n}} = 5$ when $25 < N_{k,n} \leq 50$, $Z_{N_{k,n}} = 7$ when $50 < N_{k,n} \leq 75$, $Z_{N_{k,n}} = 9$ when $75 < N_{k,n} \leq 100$, $Z_{N_{k,n}} = 11$ when $100 < N_{k,n} \leq 125$, $Z_{N_{k,n}} = 13$ when $125 < N_{k,n} \leq 150$ and $Z_{N_{k,n}} = 15$ when $150 < N_{k,n}$. A Cox Proportional hazards regression model (we used the function ‘coxph’ (Therneau and Grambsch 2000) in R) is then fitted to each treatment using only the $Z_{N_{k,n}}$ nearest neighbors. This model and the next patient’s biomarker values are used to calculate the median outcome of the next patient for both treatments. If the median cannot be found for either treatment then, the 95% lower confidence bound is used for both treatments instead.

In polynomial regression, the two binary biomarkers are used (if the function ‘coxph’ (Therneau and Grambsch 2000) in R deems them to be significant in the model) to produce a Cox Proportional hazards regression model, for each treatment. This model and the next patient’s biomarker values are used to calculate the median outcome of the next patient for both treatments. If the median cannot be found for either treatment then, the 95% lower confidence bound is used for both treatments instead.

The spline method uses the ‘sshzd’ (Chong 2014) function in R to produce ANOVA models to estimate the Cox Proportional hazards regression model for each treatment. The two biomarker values are used in the method if the function deems them to be significant in the model. The splines produced are linear and the number of knots in the model are chosen as $\max(30, 10N_{k,n}^{2/9})$ and are equally spaced. The hazard function of each treatment is calculated using the next patient’s biomarker values. The treatment with the higher predicted hazard ratio is estimated to be worse.

The Gaussian processes method uses the Gaussian processes package which does not adjust for censored data, ‘fitrgp’ (Matlab fitrgp 2016) in Matlab. We only used the data which was uncensored in the regression method, at the time each patient arrived into the study, to predict their best treatment. We used the default settings for this function.

Funding

T Jaki received funding from UK Medical Research Council (MC_UU_00002/14). This report is independent research arising in part from Prof Jaki’s Senior Research Fellowship (NIHR-SRF-2015-08-001) supported by the National Institute for Health Research. The views expressed in this publication are those of the authors and not necessarily those of the NHS, the National Institute for Health Research or the Department of Health and Social Care (DHCS). H Jackson is grateful for the support of the Engineering and Physical Sciences Research Council (Grant Number EP/L015692/1). The authors also acknowledge Quanticate for financial support and are grateful to Sarah Bowen and Karen Ooms in Quanticate for helpful discussions.

ORCID

H. Jackson http://orcid.org/0000-0003-0646-6437
T. Jaki http://orcid.org/0000-0002-1096-188X
